# Supplementary material for: We need to talk about reliability: making better use of test-retest studies for study design and interpretation
Source: PeerJ. 2019 May 24;7:e6918. doi: 10.7717/peerj.6918 (PMC6536112; doi:10.7717/peerj.6918)
Supplement: Supplemental Information 1 — The code used to perform all the calculations in the paper. [file peerj-07-6918-s001.pdf]

# Supplementary Materials: We need to talk about reliability

*Granville J. Matheson*

Below, I detail the calculations performed in the manuscript, and show the code used.

```
library(relfeas)
library(ggplot2)
library(gridExtra)
library(pwr)
```

### Example 1

**Summary:** this example shows how the reliability of a test-retest validation study with low inter-individual variance and low reliability can be approximated for an applied study with higher inter-individual variance. This shows that despite the low reliability estimated in the test-retest study, this measurement demonstrates high reliability for the research question in the applied study.

```
sd2extrapRel(sd=0.32, icc_original = 0.32,
              sd_original = 0.10)
```

```
## [1] 0.9335938
```

Even if the measurement error were to double due to the use of partial volume effect correction in the applied study (an extreme assumption), this conclusion would still hold.

```
sd2extrapRel(sd = 0.32,icc_original =  0.32,
              sd_original =  0.10, tau = 2)
```

```
## [1] 0.734375
```

**Example 2**

**Summary:** this example shows how the reliability of measurements has enormous implications for power analysis when planning a study. In this example, both measures show reliability $\geq 0.7$, but when the reliability of these measures is taken into consideration during power analysis, the minimal required sample size for this research question (more details in the paper) is nearly doubled.

```
r_attenuation(0.8, 0.7)
```

```
## [1] 0.7483315
```

```
pwr::pwr.r.test(r=sqrt(0.3), power=0.8)
```

```
##
##      approximate correlation power calculation (arctangh transformation)
##
##               n = 23.00936
##               r = 0.5477226
##       sig.level = 0.05
##           power = 0.8
##     alternative = two.sided
```

```
pwr::pwr.r.test(r=sqrt(0.3)*r_attenuation(0.8, 0.7), power=0.8)
```

```
##
##      approximate correlation power calculation (arctangh transformation)
##
##               n = 43.57966
##               r = 0.409878
##       sig.level = 0.05
##           power = 0.8
##     alternative = two.sided
```

**Example 3**

**Summary:** reliability of individual measurements, or showing the robustness of within-individual effects is often, mistakenly, taken as a proxy for good reliability of assessing between-individual differences in within-individual effects (see Hedge, Powell and Sumner, 2017). Here we demonstrate this for this particular application.

Characteristics of the sample:

```
# Sample characteristics

meanbp <- 1.91
delta_mean <- -0.12 # Mean percentage change in difference study
delta_sd <- 0.1 # SD of percentage change in difference study
sem <- icc2sem(icc = 0.8, sd =  0.22)
delta_sd_trt <- 0.073 # SD of percentage change in test-retest study



# Characteristics across two measurements
sd = delta_sd*(meanbp)
(delta_icc <- sem2icc(sem*2, sd))
```

```
## [1] -0.06137441
```

Within-individual effects:

```
# Smallest detectable difference: individual
(sdd_indiv <- 100*((sem*1.96*sqrt(2))/meanbp))
```

```
## [1] 14.27826
```

```
# Smallest detectable difference: group
samplesize <- 2
(sdd_group <- 100*(((sem/sqrt(samplesize))*1.96*sqrt(2))/meanbp))
```

```
## [1] 10.09626
```

```
# Within-individual power analysis
(power_n_within <- pwr::pwr.t.test(d= delta_mean/delta_sd_trt , sig.level = 0.05,
                                   type = "paired", alternative = "less",
                                   power=0.8))
```

```
##
##      Paired t test power calculation
##
##               n = 4.014991
##               d = -1.643836
##       sig.level = 0.05
##           power = 0.8
##     alternative = less
##
## NOTE: n is number of *pairs*
```

Between-individual assessment of within-individual changes

```
ss_total <- sumStat_total(n1 = 20,
                          mean1 =  abs(delta_mean*meanbp),
                          sd1 = delta_sd*meanbp, n2 = 20,
                          mean2 = abs(2.5*delta_mean*meanbp),
                          sd2=delta_sd*meanbp)
```

```r
# Estimation of reliability
(delta_icc_patcntrl <- sem2icc(sem*2, ss_total$sd_total))
```

```
## [1] 0.4120227
```

```r
(d_true <- ss_total$d)
```

```
## [1] 1.8
```

```r
# Using this estimated reliability for estimation of attenuation
(d_meas <- d_attenuation(rel_total = delta_icc_patcntrl, d = d_true))
```

```
## [1] 0.9509363
```

```r
# Increase in the number of required participants after taking
# reliability into account
(pwr_increase_unpaired <-
    pwr::pwr.t.test(d=d_meas, power = 0.8, alternative = "greater")$n /
    pwr::pwr.t.test(d=d_true, power = 0.8, alternative = "greater")$n )
```

```
## [1] 3.081688
```

```r
(pwr_increase_paired <-
    pwr::pwr.t.test(d=d_meas, power = 0.8, type = "paired",
                    alternative = "greater")$n /
    pwr::pwr.t.test(d=d_true, power = 0.8, type = "paired",
                    alternative = "greater")$n )
```

```
## [1] 2.281319
```

### Example 4

**Summary:** A measurement outcome with high reliability but also high variance (e.g. PBR28 for translocator protein, TSPO) is less well suited for assessing small proportional changes within individuals than a measurement outcome with low variance, even if it has relatively low reliability (e.g. AZ10419369 for frontal cortex serotonin 1B receptors). However, larger proportional within-individual changes are more likely in the former due to the larger variance.

```
tspo_hab_10es <- 10/42
ser1b_10es <- 10/6

(tspo_n <- pwr::pwr.t.test(d = tspo_hab_10es, power = 0.8)$n)
```

```
## [1] 277.8714
```

```
(ser1b_n <- pwr::pwr.t.test(d = ser1b_10es, power = 0.8)$n)
```

```
## [1] 6.760923
```

### Example 5

Variance reduction strategies (see paper) for PBR28 lead to new outcomes with poor reliability (see Matheson et al., 2017). Very large differences between groups are required before these new outcome measures begin to be reliable for applied research questions.

One can conceptualise the required Cohen's D in various ways to get an idea of whether or not such a large effect is or is not reasonable for a particular research question.

- **d** Cohen's D
- **u3** Cohen's U3
- **overlap** Distributional overlap
- **cles** Common Language Effect Size

```r
#########
# Basic #
#########

(sd_basic <- extrapRel2sd(0.7, 0.5))
```

```
## [1] 1.290994
```

```r
d_basic <- sdtot2mean2(sd_total = sd_basic, n1 = 20, n2=20, mean1 = 1)

(es_basic <- cohend_convert(d=d_basic$d))
```

```
## $d
## [1] 1.643168
##
## $u3
## [1] 0.9498259
##
## $overlap
## [1] 0.4113138
##
## $cles
## [1] 0.8773609
```

```r
##############
# Acceptable #
##############

(sd_acceptable <- extrapRel2sd(0.8, 0.5))
```

```
## [1] 1.581139
```

```r
d_acceptable <- sdtot2mean2(sd_total = sd_acceptable, n1 = 20,
                            n2=20, mean1 = 1)

(es_acceptable <- cohend_convert(d=d_acceptable$d))
```

```
## $d
## [1] 2.439262
##
## $u3
## [1] 0.9926414
##
## $overlap
## [1] 0.2226048
##
## $cles
## [1] 0.9577199
```

```r
############
# Clinical #
############

(sd_clin <- extrapRel2sd(0.9, 0.5))
```

```
## [1] 2.236068
```

```
d_clin <- sdtot2mean2(sd_total = sd_clin, n1 = 20,
                      n2=20, mean1 = 1)

(es_clin <- cohend_convert(d=d_clin$d))
```

```
## $d
## [1] 3.962323
##
## $u3
## [1] 0.9999629
##
## $overlap
## [1] 0.04757319
##
## $cles
## [1] 0.997459
```

We can also plot these effects to get a better idea of how they would look

```
graphtheme <- theme(axis.text.x=element_blank(),
                    axis.text.y=element_blank())

d_fig <- grid.arrange(
  plot_difference(d = d_basic$d) +
    labs(x='Lowest Acceptable Reliability (0.7)',
         title=NULL, y='Probability') +
    graphtheme,
  plot_difference(d = d_acceptable$d) +
    labs(x='Acceptable Reliability (0.8)',
         title=NULL, y='Probability') +
    graphtheme,
  plot_difference(d = d_clin$d) +
    labs(x='Clinical Reliability (0.9)',
         title=NULL, y='Probability') +
    graphtheme,
  nrow=1)
```

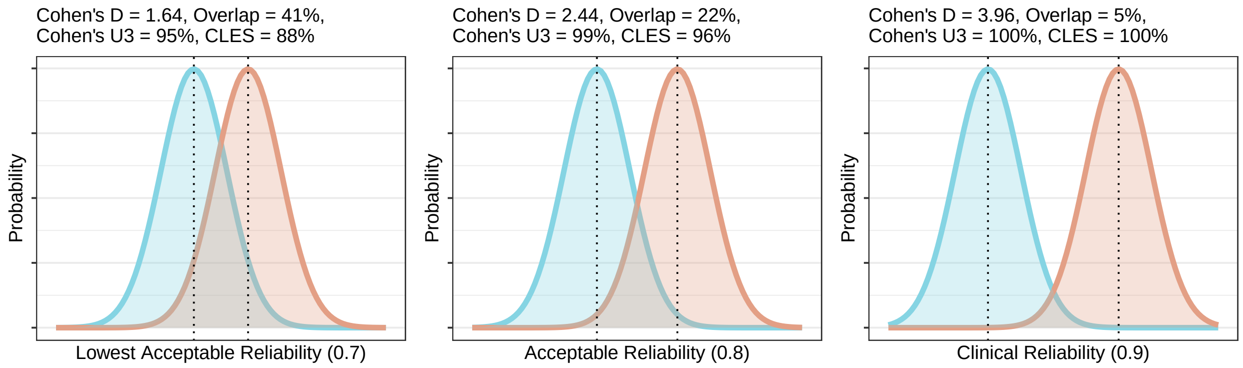


Note that the figure above, as well as the function to generate these figures and these alternative explanation metrics describing effect sizes, are inspired by the work of Kristoffer Magnusson: his interactive Cohen's D figure as well as his description of where Cohen was wrong about overlap.
